# The role of transcription factor Nrf2 in skin cells metabolism

**DOI:** 10.1007/s00403-015-1554-2

**Published:** 2015-02-24

**Authors:** Agnieszka Gęgotek, Elżbieta Skrzydlewska

**Affiliations:** Departments of Analytical Chemistry, Medical University of Bialystok, Mickiewicza 2D, 15-222 Bialystok, Poland

**Keywords:** Nrf2, Proteins expression, Reactive oxygen species, Keratinocytes, Melanocytes, Fibroblasts

## Abstract

Skin, which is a protective layer of the body, is in constant contact with physical and chemical environmental factors. Exposure of the skin to highly adverse conditions often leads to oxidative stress. Moreover, it has been observed that skin cells are also exposed to reactive oxygen species generated during cell metabolism particularly in relation to the synthesis of melanin or the metabolism in immune system cells. However, skin cells have special features that protect them against oxidative modifications including transcription factor Nrf2, which is responsible for the transcription of the antioxidant protein genes such as antioxidant enzymes, small molecular antioxidant proteins or interleukins, and multidrug response protein. In the present study, the mechanisms of Nrf2 activation have been compared in the cells forming the various layers of the skin: keratinocytes, melanocytes, and fibroblasts. The primary mechanism of control of Nrf2 activity is its binding by cytoplasmic inhibitor Keap1, while cells have also other controlling mechanisms, such as phosphorylation of Nrf2 and modifications of its activators (e.g., Maf, IKKβ) or inhibitors (e.g., Bach1, caveolae, TGF-β). Moreover, there are a number of drugs (e.g., ketoconazole) used in the pharmacotherapy of skin diseases based on the activation of Nrf2, but they may also induce oxidative stress. Therefore, it is important to look for compounds that cause a selective activation of Nrf2 particularly natural substances such as curcumin, sulforaphane, or extracts from the broccoli leaves without side effects. These findings could be helpful in the searching for new drugs for people with vitiligo or even melanoma.

## Introduction

Skin cells, being in constant contact with the surrounding environment, are highly susceptible to the effects of different stimulants. UV irradiation, xenobiotics, and thermal stress disturb cell metabolism and consequently lead to the increase in reactive oxygen species (ROS) generation and to redox imbalance. UV radiation, carrying a large dose of energy, directly converts oxygen molecules in the reactive forms and/or causes damages of the cellular macromolecules structures impairing their functions [106]. However, high or low temperatures disrupt the metabolic pathways thereby causing an overproduction of ROS that leads to the decrease in the activity of heat-labile proteins, in particular [32]. Metabolism of skin cells is also altered by xenobiotics affecting ROS generation and thereby antioxidant abilities, signal transductions, and the rate of transport through membranes [31]. All the factors, leading to an increase in ROS generation and/or a reduction in the antioxidant capacity, contribute to oxidative stress, which exposes the skin cells to the formation and accumulation of irreversibly damaged proteins, lipids, nucleic acids, and carbohydrates. This leads to a visible reduction of skin conditions, aging, and dying cells and may also induce malignant transformation [121].

## Transcription factor Nrf2

One of the ways to defend skin cells against oxidative stress is the transcriptional regulation of cytoprotectional genes by Nrf2 (Nuclear erythroid 2-related factor), in which expression in all types of epidermal cells was observed at a very high level [63]. The transcription factor Nrf2 belongs to the “cap‘n’collar” (CNC) protein family, which contains the motif called leucine zipper (bZip, basic Leucine Zipper). This family has three-dimensional structures that allow the formation of dimers with other proteins containing bZip domain. The family of transcription factors containing bZIP domain is also characterized by a basic region, which binds via hydrogen bonds to the large groove of the DNA [55].

**Fig. 1:**
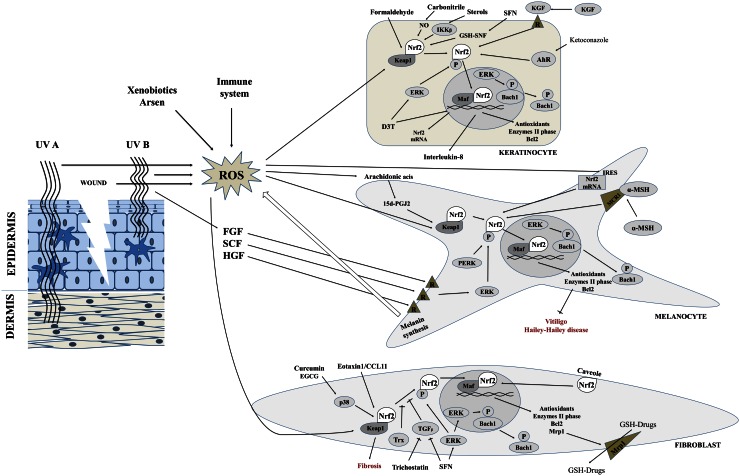
Nrf2 activation pathways in the different skin cells: keratinocyte, melanocyte, and fibroblast. *P* phosphorylation and *R* receptor

Under physiological conditions, Nrf2 encoding genes are under constant expression, as a result of which Nrf2 molecule is permanently biosynthesized. However, its level in the cytoplasm is regulated by the formation of Nrf2-Keap1-Cul3 complex [107]. Keap1 binds Nrf2 and therefore directly inhibits its activity, resulting in simultaneous Nrf2 ubiquitination catalyzed by Cul3. Binding of at least four molecules of ubiquitin to Nrf2 causes degradation of this molecule by the proteasome 26S. However, the oxidative condition in the cell leads to the oxidation of cysteine residues in Keap1 molecule, changing the conformation of the protein and causing dissociation of Nrf2 from complex [47, 82]. Free Nrf2 cannot be ubiquitinated and degraded. In turn, it is translocated to the nucleus, where it forms a complex with a small Maf protein and then is bound to the DNA in a characteristic sequence 5′-TGACnnnGCA-3′ labeled as antioxidant responsive element (ARE) and in consequence initiates the transcription of antioxidant genes (Fig. 1) [50]. Nrf2 cytoprotective action concerns mainly antioxidant enzymes such as glutathione S-transferase (GST), quinone reductase NAD(P)H (NQO1), UDP-glucuronosyltransferases (UGT), epoxide hydrolase (EPHX), γ-glutamylcysteine ligase (GCL), heme oxygenase-1 (HO-1), glutathione reductase (GR), thioredoxin reductase (TrxR), catalase (CAT), and superoxide dismutase (SOD) [76, 97, 130]. Nrf2 also activates the transcription of non-enzymatic antioxidant protein genes containing in their structure the ARE sequence (e.g., thioredoxin, ferritin) [34, 94]. The role of Nrf2 in protecting skin cells against ROS action highlights the fact that 7 % of squamous cell skin cancer in human results from mutations in Nrf2 gene [49]. Additionally, Nrf2 acts as a stimulant of anti-apoptotic proteins from Bcl-2 family [84]. The control of a wide range of antioxidants and antiapoptotic molecules causes that Nrf2 is recognized as a significant factor in the cellular response to oxidative stress, especially in the cells, which form the outer layers of the skin.

In spite of antioxidative character of Nrf2, its action may be directly modified by ROS as well as by reactive products of lipid peroxidation that influence this and cooperative proteins, particularly during oxidative stress. It was observed that low level of ROS causes the Nrf2 expression, while its high level has no effect on the Nrf2 level and leads to the irreversible cell injury and induction of apoptosis. However, it is also known that an intermediate level of ROS may participate in the control of the balance between survival and apoptosis through the activation of another transcription factor—NFκB [68, 86, 113]. Therefore, the cooperation between members of NFκB and Nrf2 pathways may exist, and the cross-talk between Nrf2 and NFκB under pathological conditions is suggested [9]. It has been shown that the Keap1/Cul3 complex could regulate both the Nrf2 and the NFκB expression through the ubiquitination. Moreover, Keap1 acts as an inducible factor for ubiquitination IKKβ, which is a cytoplasmic NFκB inhibitor. Deletion of Keap1 leads to accumulation and stabilization of IKKβ and upregulation of NFκB-derived tumor angiogenic factors [58]. On the other hand, NFκB subunits induce transcription of Nrf2 in cells at a specific promoter κB site and thus encourage resistance to chemotherapy-induced cytotoxicity [24, 101]. It was also reported that NFκB competes with Nrf2 as co-activator CREB-binding protein (CBP) [66]. Moreover, NFκB recruits histone deacetylase 3 (HDAC3) causing local hypoacetylation to hamper Nrf2 signaling [66]. However, absence of Nrf2 induces more aggressive inflammation through activation of NFκB and downstream proinflammatory cytokines [89]. Furthermore, Keap1 interacts with the NFκB-p65 subunit, thus NFκB pathway represses the Nrf2 transcriptional activity [126]. On the other hand, both NFκB and Nrf2 regulate the same group of genes, including HO-1, GCLC, Gαi2, and IL-8 [23].

Moreover, it is known that products of lipid oxidative modification generated during free radicals peroxidation as well as during enzymatic oxidation are involved in Nrf2 action. It was shown that 4-hydroxynonenal (4-HNE), one of the most reactive lipid peroxidation products, at nontoxic levels can activate stress response pathways such as Nrf2/ARE by changing Keap1 conformation [38, 109]. What is more, cell stimulation with 4-HNE at sublethal level induces adaptive response and enhances cell tolerance, primarily through induction of thioredoxin via transcriptional activation of the Nrf2 signaling pathway, thereby protecting cells against the forthcoming oxidative stress [12]. However, ROS leads to increased expression of cyclooxygenases (COX) that oxidizes arachidonic acid to PGH2 that is further metabolized by specific PG isomerases to PGE2, PGD2, PGF2α, TXA2, and prostacyclin I2 [118]. Dehydration of PGD2 leads to generation of a reactive 15d-PGJ2 that exhibits a unique spectrum of biological effects, including inhibition of IκB-kinase-β [99] and induction of glutathione S-transferase gene expression and apoptosis [53]. Moreover, 15d-PGJ2 may form adducts with Keap1 simultaneously causing dissociation of Nrf2 from complex [25]. This mechanism of Nrf2 activation was investigated in both keratinocytes and melanocytes [44, 56].

## Epidermis

The skin consists of three layers: epidermis, dermis, and hypodermis. The epidermis is the outermost layer of the skin having hydrophobic properties. It is formed mainly from keratinocytes, but it also consists dye cells—melanocytes, Langerhans cells—the cells responsible for immune reactions, and Merkel cells—cells of the nervous system.

## Nrf2 in keratinocytes

The first line of human body contact with the environment creates keratinocytes. In order to ensure skin’s resistance to external factors, the deep layers of the epidermis must have a high rate of normal cells proliferation, without disturbance in the structure and functioning. It is believed that the high resistance of the skin to external factors and its quick response to damages are related to the presence of specific receptors for growth factor (KGF—Keratinocyte Growth Factor) on the surface of keratinocytes, produced by mesenchymal cells. KGF is a small protein that can bind to the specific receptors on the keratinocytes cell membrane, which is a simultaneous signal to proliferation and to create a new layer of the epidermis at the injured place. The precise mechanism of KGF action is not completely described yet, but a direct effect of KGF on the increase of the Nrf2 activity was proposed [8]. However, Nrf2-dependent gene expression can affect the survival, differentiation, and premature death of these cells [60]. It ensures dividing the cells resistant to mutations caused by increased levels of ROS. Moreover, the increase in Nrf2 activity during keratinocytes differentiation was observed and was confirmed by the fact that in the surficial layers of the epidermis, which are the most vulnerable to external factors and are faster keratinized than younger cells, there is a higher level of antioxidant enzymes whose expression is dependent on the Nrf2 activity [60, 92].

As in other cells, in keratinocytes, ROS are generated during aerobic metabolism as well as through metabolism (mainly “respiratory burst”) of the immune system cells, which are common in the epidermis [122]. ROS, which are generated in order to protect the skin against pathogens, expose keratinocytes to depletion of antioxidant abilities and oxidative modifications of cellular components, including transcription factor Nrf2. Interactions between keratinocytes and immune system cells affect not only ROS generation, but also the efficient action of immune system [30]. Keratinocytes, as well as fibroblasts, belong to the group of cells that can produce interleukins. Interleukin-8 (IL-8) is one of the proteins whose expression is ARE dependent, and thus its level depends on the Nrf2 activity [98]. Through the release of interleukin-8 to the intercellular space, keratinocytes and fibroblasts provide communications in the whole body and fast response of the immune system to contact the harmful chemicals [127]. Increased IL-8 generation by keratinocytes is also observed in the case of mechanical skin damage. It is probably linked with the Nrf2 activation induced by oxidative stress in keratinocytes adjacent to the wound [14]. Nrf2 is also responsible for the release of others inflammatory mediators (e.g., IL-6, IL-1β, and GM-CSF) in the case of allergic contact of dermatitis. Studies of the keratinocytes line HaCaT response to allergens show that through activation of MAP kinase, Nrf2 is translocated to the nucleus. Consequently, an increase in interleukin release is observed, but the mechanism of this reaction has not yet been completely examined [74].

As ROS can interfere with the aging process and differentiation of keratinocytes, the maintenance of high levels of antioxidant enzymes (mainly HO-1, NQO1, and GST) is important for these cells. Therefore, the high activity of Nrf2 results from the activation of Nrf2, by dissociation of Nrf2-Keap1 complex, prevents deformation during keratinocyte differentiation and even malignant transformation [5, 92]. In the case of changing in level of another Nrf2 inhibitor—Bach1, which competes with Nrf2 for binding to the DNA, prevention of malignant transformation was not observed [70]. It has been shown that in Keap1-knockout mice keratinocytes, the control of transcriptional activity can be taken care of by a small protein Maf. In the case when there is no Nrf2 inhibitor, Keap1 in the cytoplasm, active, and uncontrolled Nrf2 is translocated to the nucleus, where, only after Maf-Nrf2 complex formation, it can bind DNA and initiate transcription of the genes. Therefore, in the Keap1-knockout mice keratinocytes, response to stress factors and the rate of skin aging depend on the level of Maf protein in the nucleus [79].

Disturbances in the Nrf2 activity may lead to development of various diseases. Allergic contact dermatitis (ACD) is induced usually by low molecular weight of electrophilic chemicals and metal ions, and Nrf2 is one of the key molecules that transmits a signal of disturbed redox balance and causes a biological response in dendritic cells, as well as in keratinocytes, which are in contact with them. It was shown that Nrf2 is activated by chemical sensitizers in contact dermatitis and also plays a significant role in the inflammatory immune responses [1, 48], which suggests that Nrf2 could be implicated in the chemical sensitization processes [18]. Therefore, important role of Nrf2 in controlling ACD in response to sensitizers is suggested [19]. Moreover, it is demonstrated that Nrf2 activation in keratinocytes is one of the objectives of coal tar application in case of atopic dermatitis (AD) [115]. Topical application of coal tar is one of the oldest therapies for AD. It was also found that coal tar activates the aryl hydrocarbon receptor (AhR), which can bind to the Nrf2 gene locus and increase its expression [116]. As a result, higher Nrf2 level leads to induction of NQO1 transcription [40, 116].

## Extracellular Nrf2 activators in keratinocytes

Oxidative stress in keratinocytes may be generated by xenobiotics, e.g., arsenic, which is an inducer of carcinogenesis in HaCaT cell line. Increased ROS generation (mainly hydrogen peroxide) lead to the increase in Nrf2 expression, at transcription and translation level, as well as the accumulation of active Nrf2 in the nucleus of those cells [91]. Xenobiotics strongly sensitizing skin such as formaldehyde, eugenol, or dinitrochlorobenzene elicit the skin’s defences through Nrf2 activation. As a result of covalent links between these compounds and the cysteine residues in Keap1, Nrf2 dissociates from Nrf2-Keap1-Cul3 complex and consequently begins the ARE-dependent gene expression [10, 78]. Many chemopreventive phytochemicals are known to activate Nrf2 either by oxidative or covalent modification of its cytosolic repressor—Keap1 or by phosphorylation of Nrf2 [15].

Nrf2 also protects skin cells from UV radiation. Overexpression of the Nrf2 gene in mice skin keratinocytes exposed to UVB radiation causes higher resistance to apoptosis [54]. Incubation of the keratinocytes line HaCaT with flavonoids, such as quercetin or kaempferol, significantly protects cells against UV radiation with the increase of Nrf2 level in the cytoplasm and cells viability [45, 51]. Therefore, there is a constant search for a highly selective activator of Nrf2 in keratinocytes that not induce side effects. A promising compound of natural origin is sulforaphane (SFN) isolated mainly from cruciferous plants such as broccoli or brussels sprouts [103]. Mechanism of SFN action involves a reduction in the GSH level, which in turn alters the Keap1 conformation and its inhibitory properties, and consequently the active Nrf2 is released into the cytoplasm [80, 120] and enhances the expression of antioxidant enzymes (NQO1, HO-1, γGCS) in keratinocyte line HaCaT [121]. The extract containing the SFN reduces the risk of carcinogenesis induced by UV radiation in mice line SKH-1 [16]. Moreover, the above-mentioned extract given to animals with benign tumor of the skin reduced the tumor weight [104]. However, studies conducted on volunteers subjected to UV light and treated with SFN showed a decrease in the development of skin erythema up to 90 % [111].

Nrf2 activity may also be indirectly affected by the plant sterols (e.g., (Z)-guglesterone). Their action is associated with the activation of IκB, whereby its physiological activator IKKβ may remain in an inactive form and bind to Keap1, thereby blocking Nrf2 ubiquitination and increasing the level of active Nrf2 in cells [2]. Other compounds that could activate Nrf2 are carbonitriles whose metabolism increased NO level and can lead to nitrosylation of Keap1 cysteine’s. This modification alters the conformation of Keap1 and leads to a release of Nrf2 into the cytoplasm [65]. Another natural compound that enhances Nrf2 mRNA level and phosphorylation by ERK kinases that leads to increase in the transcriptional Nrf2 activity is D3T (3H-1,2-dithiole-3-thione) [57, 72]. It is also suggested that dietary supplements containing ellagic acid are based on the activation of Nrf2. It was shown that ellagic acid results in a higher cells survival after UVA radiation. Furthermore, these cells exhibited a higher resistance to ROS generation and cellular components oxidative modifications, which may be associated with increased expression of antioxidant enzymes (HO-1 and SOD) [36].

The mechanism of certain drugs action on epidermal keratinocytes (NHEK) is also associated with Nrf2 activation [116]. Ketoconazole, an antifungal agent from the group of azoles, activates the cytoplasmic receptor AhR and forms with them active transcription complex, which is translocated to the nucleus where it binds DNA and initiates gene expression [11, 69]. It is directly related to the increase in the transcriptional Nrf2 activity, and therefore a reduction of the inflammatory response but the exact mechanism of the intersection of these two pathways is not fully understood yet [52].

## Nrf2 in melanocytes

Except the keratinocytes, the epidermal layer includes also melanocytes. They are distributed mainly near the basal membrane of the epidermis. Melanocytes are small cells with a low content in the central and have numerous long cytoplasmic appendixes, which penetrates the layers of keratinocytes. There are two types of beans stored inside these appendixes: melanosomes—capable to synthesizing melanin and melanin grains. Melanin is responsible for the dark pigmentation of human skin, thereby protecting the deeper layers of the skin from UV radiation that is also a stimulator of the melanin synthesis [85]. During melanogenesis, tyrosinase, the major tyrosine metabolism enzyme, may show diphenolaze (H_2_O_2_ generation) or catalase (H_2_O_2_ decomposition) activity. Therefore, the synthesis of melanin may be associated with higher ROS generation [112]. Nrf2 activity protects melanocytes against the harmful ROS effects. It has been shown that overexpression of Nrf2 caused by transfection of plasmids containing the Nrf2 gene (pCMV6-XL5) or Keap1 mRNA silencing using siRNA prevents oxidative stress induced by xenobiotics in melanocytes cell line PIG1 or NHK [41, 73]. Furthermore, ex vivo studies have shown that enhanced level/activity of Nrf2 and protein whose synthesis is dependent on this factor reduces the effects of oxidative stress formed after exposure to UVB radiation. Nrf2 activation mechanism in melanocytes is associated with a higher level of melanotropine (α-MSH)—the hormone produced in the pituitary gland, whose binding to a specific receptor on the surface of melanocytes (MC-1R), leads to the formation of complexes initiating ARE-dependent genes transcription [54]. Other studies show that the increased level of active Nrf2 is directly related to the IRES sequence (internal ribosome entry sequence) contained in the Nrf2 transcript, which is responsible for transcription-dependent redox state [95]. This transcript receives a signal from the cytoplasm about the unbalanced redox status and begins synthesis of the new Nrf2 molecules [104].

There is a strong evidence suggesting influence of the degree of phosphorylation on the Nrf2 activity. As a result of oxidative stress, Nrf2 is dissociated from Keap1-Nrf2 complex and then as a free molecule can be phosphorylated. On the other hand, it is known that expression of many kinases (mainly MAPK family, PI3K) is increased during oxidative stress; therefore, the level of phosphorylated Nrf2 is also rapidly increased [108]. Moreover, Nrf2 is phosphorylated by kinase ERK1/2 activated on Ras/Raf/MEK/ERK signaling pathway [59]. On the other hand, ERK activation in nucleus can lead to phosphorylation of Bach1—protein, which under physiological conditions binds DNA in a sequence of ARE, blocking the Nrf2 activity. Phosphorylated Bach1 loses the ability to bind to DNA and allows Nrf2 to start the transcription and antioxidant protein synthesis, whereby cells become highly resistant to the oxidative stress induced by UV radiation [129]. Additionally, the level of phosphorylated Nrf2 is increased by phenolic compounds that activate the PERK kinase [114].

The number of melanocytes in the skin of people of different races is similar, and the differences in color are only due to the intensity of the melanin production. It is estimated that the epidermal ratio of melanocytes to keratinocytes is around 1–40 (depending on the part of the body). After exposure to UV radiation, the amount and the activity of the melanocytes are regulated by keratinocytes through the synthesis and release of signaling compounds (e.g., FGF, SCF, HGF) into the intercellular space [35]. These molecules are paracrine growth factors, and after binding one of them to specific receptors (FGFR1/2, c-kit, c-Met), the activation of signaling cascade pathway is responsible for cell proliferation, differentiation, and motility, as well as the initiation of intensity of melanin synthesis [17].

Melanocytes, during the whole period of life, retain the ability to proliferate, as a result of adaptation to changing environmental conditions associated with the intensity of solar radiation throughout the year [27]. Furthermore, the melanocytes are highly sensitive to apoptosis caused by chemical signals caused by bacterial toxins, microtubule structure damaging substances, or protein synthesis inhibitors [37]; therefore, their reduction must be complemented by continuously proliferating cells. However, generated during proliferation and continuously accumulated errors in the genome lead to uncontrolled proliferation of cells and start the process of carcinogenesis, which leads to the development of malignant melanoma [39]. In addition, the constant oxidative stress associated with exposure to radiation, changes in temperature, and the effect of xenobiotics cause the accumulation of oxidative damages in these cells. Therefore, high level of Nrf2 synthesis is constantly maintained in melanocytes [41]. Studies show that disturbances in the synthesis or activation of Nrf2 reduce the resistance of cells to stress, both physical and chemical, leading to cell death or to carcinogenesis [42]. Regardless of the influence of above factors, Nrf2 activity is also dependent on the Maf—nuclear protein level and chemical structure that may be affected by viral infections of the skin. This causes an increase in the intensity of Nrf2-Maf transcription complex formation and their strength of DNA binding, which can lead to uncontrolled antiapoptotic protein overexpression and consequently even to the process of carcinogenesis [62].

## Extracellular Nrf2 activators in melanocytes

Nrf2 is also involved in response of the skin to many diseases, e.g., in the case of Hailey–Hailey disease (bullous disease, HHD); subcutaneous injection of afamelanotide causes the increase of active Nrf2 level in melanocytes and keratinocytes that result in a reduction in the level of ROS and local inflammation [7]. Activation of Nrf2 may also be associated with genetic anomalies. It has been shown that the incidence of vitiligo in humans depends on an Nrf2 gene set. In people with vitiligo, significantly lower levels of Nrf2 m-RNA compared to healthy subjects were reported [3]. Depending on the activity of newly generated Nrf2, resistance of melanocytes to oxidative stress and the risk of vitiligo are changed [26, 42]. Furthermore, in melanocytes from patients treated with curcumin, a strong increase in phase II enzymes synthesis is observed, but simultaneously it results in increase in the apoptosis in the keratinocytes [81].

## Dermis

Dermis mainly consists of fibroblasts, which are located between the connective tissue (collagen and elastin fibers), nerves, and blood vessels. These cells are responsible for the synthesis and secretion of collagen, elastin, hyaluronic acid, or glycosaminoglycans into the intercellular space, thus providing strength and elasticity of the skin. Being in the middle layer of the skin, fibroblasts are not directly (as keratinocytes) exposed to the environmental factors. These cells during the whole life have a possibility to proliferate, especially in case of damage of the dermis, but unfortunately with age their activity slows down. This is accompanied by a reduction in metabolic capacity and decrease in the rate of replication, which causes the weakening and the disappearance of the skin-supporting elements. According to the free radical theory of aging, these changes are attributed to ROS action.

## Nrf2 in fibroblasts

Under physiological conditions, Nrf2 controls the proper functioning of the fibroblasts. Studies on mouse embryonic fibroblasts (MEFs) show that knockdown of Nrf2 genes expression leads to a reduction in glutathione levels up to 80 % relative to wild-type cells [33]. In consequence, knockdown of Nrf2 genes expression in mice fibroblasts significantly reduces their resistance to oxidative stress and survival [43], and fibroblasts derived from Nrf2 knockout mice also exhibit a much lower resistance to oxidative stress as compared to cells derived from control animals [130].

Because of epidermal layer, UVB radiation does not reach the dermis, but fibroblasts still can be exposed to UVA. Experiments on fibroblasts show that in these cells the Nrf2 activation occurs in varying degrees after exposure to different UVA wavelengths that induce a strong immune response, simultaneously leading to the transcription of many phase II antioxidant enzymes [75]. However, UVB radiation does not cause such a reaction, thereby leading to DNA damage and apoptosis, and the link between Keap1-Nrf2 pathway and apoptosis in fibroblasts was shown [46]. Tests on the mouse fibroblasts line L929 showed that H_2_O_2_-induced oxidative stress leads to the activation of Nrf2 and induction of antioxidant gene expression, as well as to increase in the level of anti-apoptotic proteins from Bcl-2 family [46]. It was shown that fibroblasts with *Bcl*-*2* gene silencing and fibroblasts incubated with an inhibitor of Bcl-2 protein (HA14-1) have reduced level of active Nrf2 [67]. However, the inhibitor of Keap1-Cul3 complex formation affects the process of apoptosis by binding to Bcl-2 and its ubiquitination that reduces the antiapoptotical potential of cells [83].

Fibroblasts are characterized by two different mechanisms for the inactivation of the transcription factor Nrf2. Except for the cytoplasmic inhibitor, Keap1, fibroblasts also have a second mechanism of binding and inactivation of Nrf2 using a caveolae that are a vesicular structures formed by a dent fibroblast cell membranes [117]. They are also observed in adipocytes and endothelial cells, and their main function is to participate in membrane trafficking and endocytosis [100]. Therefore, it is believed that they can also take part in the degradation of factor Nrf2, but the exact mechanism of Nrf2-caveolae interaction has not yet been elucidated [64]. Dual mechanism of Nrf2 binding existing in fibroblasts probably allows cells to increase the pool of this factor in the cytoplasm under physiological conditions and thus to faster and stronger immune response to oxidative stress conditions.

Fibroblasts are cells able to differentiate. Cell culture studies suggest that inhibition of Nrf2 activity by treating cells with TGF-β leads to an increase in the level of ROS that can cause fibrosis and fibroblast differentiation to miofibrocytes [121]. However, the level of endogenous TGF-β as well as fibrosis process can be inhibited by Nrf2 activators such as SFN or trichostan that enhance Nrf2 binding to DNA [128]. The Nrf2 level/activity may also be affected by hormonal signaling molecules including ERRα (estrogen-related receptor α), but the mechanism of their interaction has not been found yet [96]. One of the better-known Nrf2 activation mechanisms used in the treatment of skin diseases is the action of curcumin. Therefore, curcumin, a turmeric root extract, has been demonstrated to induce antifibrotic cell activity. Curcumin disturbs the TGF-β signaling in systemic scleroderma (SSc), by counteracted phosphorylation of Smad2 and induced upregulation of TGF-β-induced factor (TGIF)—a negative regulator of TGF-β signaling. Moreover, curcumin-mediated Nrf2 activation leads to a decrease in the level of ROS that can cause suppression of fibrotic process in scleroderma [110, 122, 123].

## Nrf2 activators in fibroblasts

The activity of fibroblast Nrf2 is also reduced by thiol antioxidants such as thioredoxin that free thiol group may prevent Keap1 oxidation, which favors the maintenance of Nrf2 in Keap1 complex. However, during oxidative stress, the level of antioxidants including thioredoxin is reduced, and its effect on Nrf2 is abolished [90]. Other natural compounds that affect the activity of Nrf2 are eotaxins. Eotaxin-1/CCL11 is a natural chemokine, which appears in the intracellular matrix as a response to occurrence of stress. This chemokine increases the activity of Nrf2 in cultured fibroblasts [22], while in skin cells, patients with atopic skin eotaxin-1/CCL11 level are reduced which is contributed to the reduced activity of Nrf2 and decreased antioxidant skin cells capacity [88].

The Nrf2 activity is also involved in Mrp family expression (multidrug resistance-associated proteins) [71]. Mrp proteins are ATP-dependent membrane transporters, and their main function is to remove, from the cell, glutathione conjugates with harmful substances—mainly metabolites of drugs [61]. The highest Mrp level is noted in the hepatocytes, but in the skin fibroblasts, the level of these proteins is also high [87]. Studies on fibroblasts isolated from Nrf2 knockout mice (−/−) show that MRP1 transcript level in these cells was significantly lower compared to the control cells. Moreover, in fibroblasts Nrf2 (+/+) treated with diethyl maleate, increase in the Mrp1 level was observed, while in the case of fibroblasts Nrf2 (−/−), there was no such reaction [29]. Those results indicate that Nrf2 in fibroblasts has influence on both constitutive and inducible Mrp family expression.

The fibroblasts Nrf2 activity is also modified by many others natural compounds including polyphenols, such as curcumin, EGCG (epigallocatechin-3-gallate) or apomorphine, and flavone derivatives as well as components of pepper betle, brassica plants, and walnut sprouts extracts [28]. Polyphenols affect Nrf2 activity by effecting signaling pathways associated with p38, B-Raf, and NF-κB [4, 77]. However, flavone derivatives enhance Nrf2 level/activity by increase in fibroblast line NIH3T3 Nrf2 mRNA level and in active Nrf2 via activation of ERK1/2 [21, 102]. Natural Nrf2 activators are also found in the brassica plants. One of these compounds which increases the activity of Nrf2 and not causes toxic effects on NIH3T3 cells is 3,3-diindolylometan which is derived from indol-3-carbinol fermentation [20]. Walnut sprouts extracts also contain natural Nrf2 activators that cause an increase in cells resistance to oxidative stress and increase survival up to 50 % in the case of fibroblast cells exposed to UVB [13].

## Uncontrolled activation of Nrf2 in skin cells

Most of the results suggest rather beneficial effects of Nrf2 activation under physiological conditions. However, Nrf2 activity is inhibited by Keap1, and deletion of this second protein gene in mice caused death of these animals within the first 3 weeks after birth due to hyperkeratosis in the esophagus and stomach, resulting in nutrient obstruction and stomach ulceration. These mice also revealed severe scaling and hyperthickening of the cornified layer of the epidermis [79, 119]. It was also shown that chronic Nrf2 activation causes sebaceous gland enlargement and seborrhea in mice keratinocytes due to upregulation of the growth factor epigen, which was identified as a novel Nrf2 target [105]. It was accompanied by thickening and hyperkeratosis of hair follicle infundibula. These abnormalities caused dilatation of infundibula, hair loss, and cyst development upon aging. Upregulation of epigen, secretory leukocyte peptidase inhibitor (Slpi), and small proline-rich protein 2d (Sprr2d) in hair follicles was identified as the likely cause of infundibular acanthosis, hyperkeratosis, and cyst formation. These alterations were highly reminiscent to the phenotype of metabolizing acquired dioxin-induced skin hamartomas (MADISH) patients. Indeed, Slpi, Sprr2d, and epigen were strongly expressed in cysts of MADISH patients and upregulated by dioxin in human keratinocytes in an Nrf2-dependent manner. These results identify novel Nrf2 activity in the pilosebaceous unit and a role of Nrf2 in MADISH pathogenesis [105]. Other findings suggest that the constitutive activation of Nrf2 in the epidermis and its binding to the promoters of differentiation-specific genes in keratinocytes may lead to abnormal enhancement of keratinocytes [6].

## Summary

The protection of proper skin functions needs cooperation of different mechanisms. One of them protects cellular components against oxidative damages by antioxidant proteins biosynthesis which is dependent on transcription factor Nrf2 activity (Fig. 1). The increase in the activity of Nrf2 enhances cell resistance to oxidative stress caused by UV and chemicals and in consequence could prevent malignant transformation. Nrf2, especially in keratinocytes and melanocytes, protects these cells against mutation during process of keratinization and melanogenesis. However, fibroblast Nrf2 plays an important role in protection of these cells against differentiation and fibrosis. Moreover, Nrf2 participation in wound healing and inflammation inhibition is also essential for maintaining the integrity of the skin.
